# Effects of Gas Composition on the Lipid Oxidation and Fatty Acid Concentration of Tilapia Fillets Treated with In-Package Atmospheric Cold Plasma

**DOI:** 10.3390/foods13010165

**Published:** 2024-01-03

**Authors:** Xiaohan Sang, Yuanyuan Wang, Jiamei Wang, Zhicheng Cai, Lixian Zeng, Wentao Deng, Jianhao Zhang, Zhumao Jiang

**Affiliations:** 1 Hainan Engineering Research Center of Aquatic Resources Efficient Utilization in South China Sea, Key Laboratory of Seafood Processing of Haikou, School of Food Science and Engineering, Hainan University, Haikou 570228, China; 17861120582@163.com (X.S.); yuanyuanwang0224@163.com (Y.W.); czc@hainanu.edu.cn (Z.C.); zeng18218841215@163.com (L.Z.); w18316478401@163.com (W.D.); 2 College of Food Science and Technology, Nanjing Agricultural University, Nanjing 210014, China; nau_zjh@njau.edu.cn; 3 College of Life Sciences, Yantai University, Yantai 264005, China; jiangzhumao@hotmail.com

**Keywords:** cold plasma, lipid oxidation, tilapia fillets, gas composition, fatty acid

## Abstract

Cold plasma (CP) is a non-thermal preservation technology that has been successfully used to decontaminate and extend the shelf life of aquatic products. However, the preservation effect of CP treatment is determined by several factors, including voltage, time, and gas compositions. Therefore, this study aimed to investigate the effects of gas composition (GasA: 10% O_2_, 50% N_2_, 40% CO_2_; GasB: air; GasC: 30% O_2_, 30% N_2_, 40% CO_2_) on the lipid oxidation of tilapia fillets treated after CP treatment. Changes in the lipid oxidation values, the percentages of fatty acids, and sensory scores were studied during 8 d of refrigerator storage. The results showed that the CP treatment significantly increased all the primary and secondary lipid oxidation values measured in this study, as well as the percentages of saturated fatty acids, but decreased the percentages of unsaturated fatty acids, especially polyunsaturated fatty acids. The lipid oxidation values were significantly increased in the GasC-CP group. After 8 d, clearly increased percentages of saturated fatty acids, a low level of major polyunsaturated fatty acids (especially linoleic (C18:2n-6)), and a decrease in the percentages of eicosapentaenoic acid (C20:5n-3) and docosahexaenoic acid (C22:6n-3) were found in GasC-CP; that is, the serious oxidation of lipids was found in the high O_2_ concentration group. In addition, the sensory score was also lower than that of the hypoxia CP group. Therefore, high O_2_ concentrations can enhance lipid oxidation and the changes in the fatty acid concentration. Controlling the O_2_ concentration is reasonable to limit the degree to which lipids are oxidized in tilapia after the in-package CP treatment.

## 1. Introduction

Tilapia is a global aquaculture fish species that has broad application prospects due to its high nutritional value, abundance of essential amino acids, especially lysine, tender meat, unique flavor, and low price [1,2]. However, owing to its high water and nutrient content, tilapia is easily affected by microorganisms and endogenous enzymes, and it is prone to spoilage during storage [3]. With consumers paying more and more attention to food quality and safety, it is very important to maintain freshness and extend the shelf life of tilapia during storage. The traditional preservation methods commonly used in aquatic products include low-temperature storage, ice preservation, partially frozen storage, and so on [4]. However, they generally have the problems of high transportation costs and a short preservation period, with the structural characteristics of the fish itself often being destroyed during frozen storage [5]. Recently, cold plasma (CP), as a new non-thermal processing preservation technology, has been found to kill a large number of microorganisms, inhibit the deterioration of aquatic products while retaining the original quality of aquatic products, and prolong their shelf life, so it is widely used in aquatic product preservation.

CP induces the generation of many reactive species, such as electrons, positive and negative ions, molecules (ground or excited state), reactive oxygen and nitrogen species (ROS and RNS, respectively), and electromagnetic radiation (ultraviolet and visible light) [6]. Among them, ROS produced by CP, namely ozone (O_3_), hydroxyl radicals (·OH), hydrogen peroxide (H_2_O_2_), and other oxygen-containing reactive groups, readily oxidize unsaturated fatty acid double bonds and trigger a series of corresponding lipid autoxidation reactions [7,8]. Numerous investigations have demonstrated the ability of CP to accelerate treatment-induced lipid oxidation in fish, including Atlantic herring [5], Asian sea bass [9], mackerel [10], golden pompano [4], and other fish. As such, CP treatment has adverse effects on fish lipids, and it is therefore crucial to control the lipid oxidation caused by CP treatment.

The excite gas composition and content are the predominant determinants of the reactive species generated in CP; the degree of lipid oxidation in food treated with CP is different due to the different gas compositions. Asian sea bass slices preserved in CP comprising a 90:10 ratio of Ar and O_2_ (% *w*/*w*) presented lower lipid oxidation values during storage than those treated with 60% CO_2_, 30% Ar_2_, and 10% O_2_. Evidently, the elevated CO_2_ concentration triggered carbonic acid-mediated fish protein denaturation and the release of free heme iron, which promoted lipid oxidation in Asian sea bass meat [9]. In a study on the lipid oxidation of Pacific white shrimp using CP generated by mixed gases of Ar/Air (80:20) and Ar/O_2_ (80:20), after 15 d of refrigerator storage, Ar/air-generated CP induced a gradual increase in thiobarbituric acid reactive substances (TBARS) and saturated fatty acids and the decomposition of unsaturated fatty acids [11]. Similar alterations were also observed in animal meat. In Hamburg (containing 60% beef) [12] and pork [13], CP treatment with different gas compositions yielded distinct lipid oxidation values. Therefore, variations in the gas composition variably affect lipid oxidation in CP-treated food, and regulating the combination of gases is pivotal for mitigating lipid oxidation in food.

Our team has studied the microbial state of tilapia fillets during refrigerator storage under CP treatment conditions of 70 kV and 3 min [14]. The results show that the total number of tilapia fillets in the CP treatment group is significantly lower (*p <* 0.05) than that in the untreated group during refrigerator storage for 0–8 d, and the microbial stability is high. Therefore, on the premise of prolonging the shelf life of tilapia fillets, we explored the changes in lipid oxidation and fatty acid concentration by optimizing the conditions of CP treatment in order to reduce its influence on the lipid oxidation of tilapia fillets. In this study, tilapia fillets were packaged in GasA (10% O_2_, 50% N_2_, 40% CO_2_), GasB (air), and GasC (30% O_2_, 30% N_2_, 40% CO_2_) and treated using a dielectric barrier discharge (DBD) CP system. Then, the variations in the contents of primary and secondary lipid oxidation products, fatty acid concentrations, and sensory evaluation were analyzed after treatment to explore the effects of gas composition on lipid oxidation and fatty acid. The results of this study could provide theoretical support to facilitate the application of CP in the preservation of tilapia and aquatic products, as well as to regulate the composition of CP treatment gases in order to delay lipid oxidation.

## 2. Materials and Methods

### 2.1. Sample Preparation

Fresh tilapia fillets were purchased from Hainan Quan Yi Food Company Ltd., Haikou, China.

The fillets were put in a sturdy polypropylene container (210 mm × 133 mm × 35 mm) after dividing them into three groups. The containers were evacuated of air using a gas packaging machine (MAP-H360, Suzhou Senry Fresh Equipment Company Ltd., Suzhou, China). The three groups were sealed with gases as follows: GasA (10% O_2_, 50% N_2_, 40% CO_2_), GasB (air), and GasC (30% O_2_, 30% N_2_, 40% CO_2_).

### 2.2. CP Treatment

The DBD system used in this study was the same as that described by Wang et al. [14]. The sealed package samples were treated directly at 70 kV for 3 min. After treatment, they were stored at 4 °C for 8 d, and samples were taken every 2 d. The samples without CP treatment were taken at the same time as the controls.

### 2.3. Sensory Analysis

Sensory training was conducted prior to the experiments according to the standard “Guidelines for sensory evaluation of aquatic products-GB/T 37062 2018” [15]. Several representative standards about the characteristics of tilapia fillets were collected, and sensory evaluators were required to describe and evaluate them independently. After that, sensory evaluation was conducted by 10 well-trained and experienced team members (5 males and 5 females, aged 22–29 years) in the sensory analysis room (25 °C). On each sampling day, each group of 3 fish fillets was packed in a box marked with a 3-digit code and presented to each group member blindly in random order. By using a 5-point (5 to 1) descriptive scale (Table S1), the team members rated the brightness, color, texture, muscle elasticity, and odor of the samples (less than 3 is considered unacceptable) [16], and the results were expressed as the mean score for each attribute. The radar maps were drawn to display the sensory evaluation results.

### 2.4. Lipid Extraction

The lipids in the tilapia fillets were extracted according to the methods proposed by Folch et al. [17], with slight modifications. The minced fish muscle (1 g) was mixed with 5 mL of chloroform/methanol (2:1, *v*/*v*). After shaking at room temperature for 4 h, 2.25 mL of potassium chloride solution (0.88%, *w*/*t*) was added to the mixture, and shaking was continued at room temperature for 30 min. Subsequently, the mixture was filtered and centrifuged to filtrate with a high-speed freezing centrifuge (TGL-1650, Sichuan ShuKe Instrument Co., Ltd., Chengdu, China) at 4000 r/min for 10 min to collect the lower phase in the tube. Finally, the organic reagents in the lipid solution were evaporated with nitrogen (high purity, 99.99%, 60 mL/min) to obtain the concentrated lipids. The lipids were stored at −20 °C for further analysis.

### 2.5. Lipid Oxidation Analysis

#### 2.5.1. Peroxide Value (POV)

The POV values were determined according to the method described by Ke et al. [18]. First, the lipids (1 g) were dissolved in 30 mL of chloroform/acetic acid solution (2:1, *v*/*v*); then, 1 mL of saturated potassium iodide solution was transferred and incubated in the dark for 3 min. After that, 100 mL of deionized water was added to the mixture and shaken thoroughly. Then, the mixture was titrated with sodium thiosulfate solution (0.01 mol/L) until its color turned yellow. Subsequently, starch indicator (1%, *w*/*v*) (1 mL) was added, and then the titration was continued until the blue disappeared. A blank test without lipids was carried out at the same time. The results were calculated as g/100 g using the following equation:

$$POV=\frac{(V-V_{0})\times 0.01\times 0.1269}{m}\times 100$$

where *V*_0_ and *V* are the volumes of sodium thiosulfate solution titrated for the blank and test samples (mL), and *m* is the weight of the sample (g).

#### 2.5.2. Conjugated Diene Value (CDV)

The method proposed by Yuan et al. [19] was adopted for CDV, with slight modifications. The lipids (1 g) were mixed with 25 mL of isooctane. After sufficient shaking to dissolve, the solution was set in the dark for 10 min. Then, the absorbance at 232 nm was measured with an ultraviolet spectrophotometer (T6 New Century, Beijing Puxi General Instrument Co., Ltd., Beijing, China). The CDV value was calculated using the following formula:

$$CDV=\frac{A_{232}}{c\times l}$$

where *c* is the sample concentration (g/mL), and l is the length of the Shi Ying cuvette (1 cm).

#### 2.5.3. Thiobarbituric Acid Reactive Substances (TBARS)

The TBARS were measured according to the method proposed by Du et al. [20], with slightly modified modifications. Minced fish (5 g) was homogenized with 50 mL of 7.5% (*w*/*v*) trichloroacetic acid (containing 0.1% EDTA) for 1 min at 6000 r/min. After shaking at 50 °C for 30 min, it was centrifuged at 6000 r/min for 5 min. The supernatant (5 mL) was mixed with thiobarbituric acid (20 mmol/L, 5 mL) solution and, at the same time, another 5 mL of trichloroacetic acid was mixed with thiobarbituric acid solution instead of the supernatant as a sample blank. The mixture was incubated in a water bath at 90 °C for 30 min and cooled with flowing water. The absorbance of the solution was measured at 532 nm. The TBARS value was calculated according to the standard curve of malondialdehyde (MDA), and the results were expressed as milligrams of MDA/kg of fish sample.

#### 2.5.4. Acid Value (AV)

The determination of AV followed the method proposed by Zhang et al. [21]. Briefly, the lipid samples (1 g) were dissolved in 30 mL of isopropanol/ether solution (1:1, *v*/*v*) with three drops of phenolphthalein indicator and fully mixed. Then, the mixture was titrated with sodium thiosulfate solution (0.01 mol/L) until its color appeared slightly red at first and without any significant fading within 15 s. The blank test without a lipid sample was carried out. The result was calculated using the following formula:

$$AV=\frac{(V_{1}-V_{2})\times 0.01\times 56.1}{m}$$

where *V*_2_ and *V*_1_ are the volumes of sodium thiosulfate solution titrated for the blank and test samples (mL), and *m* is the weight of the test sample (g).

#### 2.5.5. P-Anisidine Value (*p*-AV)

The P-anisidine value was determined by using the method of Jadhav et al. [22], with slight modifications. The lipids were dissolved in isooctane with a ratio of 1:100 (*w*/*v*); then, the samples (5 mL) and isooctane (5 mL) were mixed with 1 mL of p-anisidine reagent (0.0125 g of methoxyaniline in 50 mL of acetic acid) and incubated in the dark for 10 min. After that, the absorbances of *A*_1_ and *A*_2_ were measured at 350 nm, respectively. In another 5 mL sample, 1 mL of acetic acid was added, and the absorbance was measured as *A*_3_ at 350 nm. The *p*-AV value was calculated using the following formula:

$$p-AV=\frac{100\times c_{1}\times V}{m}\times (A_{1}-A_{2}-A_{3})\times 1.2$$

where *c*_1_ is the sample concentration (g/mL), *V* is the sample volume (25 mL), and *m* is the weight of the test sample (g).

#### 2.5.6. Total Oxidation Value (TOTOX)

Using the approach that Chen et al. [23] outlined, the TOTOX value was calculated as follows:

TOTOX = (4 × POV) + *p*-AV

### 2.6. Fatty Acid Analysis

The method of Wang et al. [24] was used and slightly modified. Methyl esterification: the lipid samples were reacted with 2 mL of methanol sulfate (1%, *v*/*v*) at 70 °C for 30 min and cooled to 25 °C. Then, 1 mL of n-hexane solution and distilled water were added, shaken for 1 min, and allowed to stand to obtain a layered solution. The upper organic layer was aspirated, and the solvent was evaporated. Before analysis, the sample passed through an organic filter membrane (0.22 μm).

GC conditions: GC-FID (7890A Agilent, Palo Alto, CA, USA) and CP-Sil 88 capillary columns (100 m × 0.25 mm, 0.20 μm film thickness) equipped with a hydrogen ion flame detector were used. The heating program was as follows: the temperature was maintained at 45 °C for 4 min, then increased to 175 °C at 13 °C/min for 27 min, and then increased to 215 °C at 4 °C/min for 35 min. According to the mass spectrum search, the detected fatty acid components were qualitatively analyzed, and then the percentage content of each fatty acid was calculated using the peak area normalization method.

### 2.7. Statistical Analysis

The data were analyzed using SPSS 20.0 (SPSS Inc., Chicago, IL, USA) for one-way variance (ANOVA) and checked for significance using Duncan’s multiple comparison test (*p* < 0.05). The data were plotted using Origin 2019. Each experiment was repeated three times (*n* = 3). The data were expressed as the mean ± standard deviation.

## 3. Results and Discussion

### 3.1. Sensory Analysis

**Figure 1:**
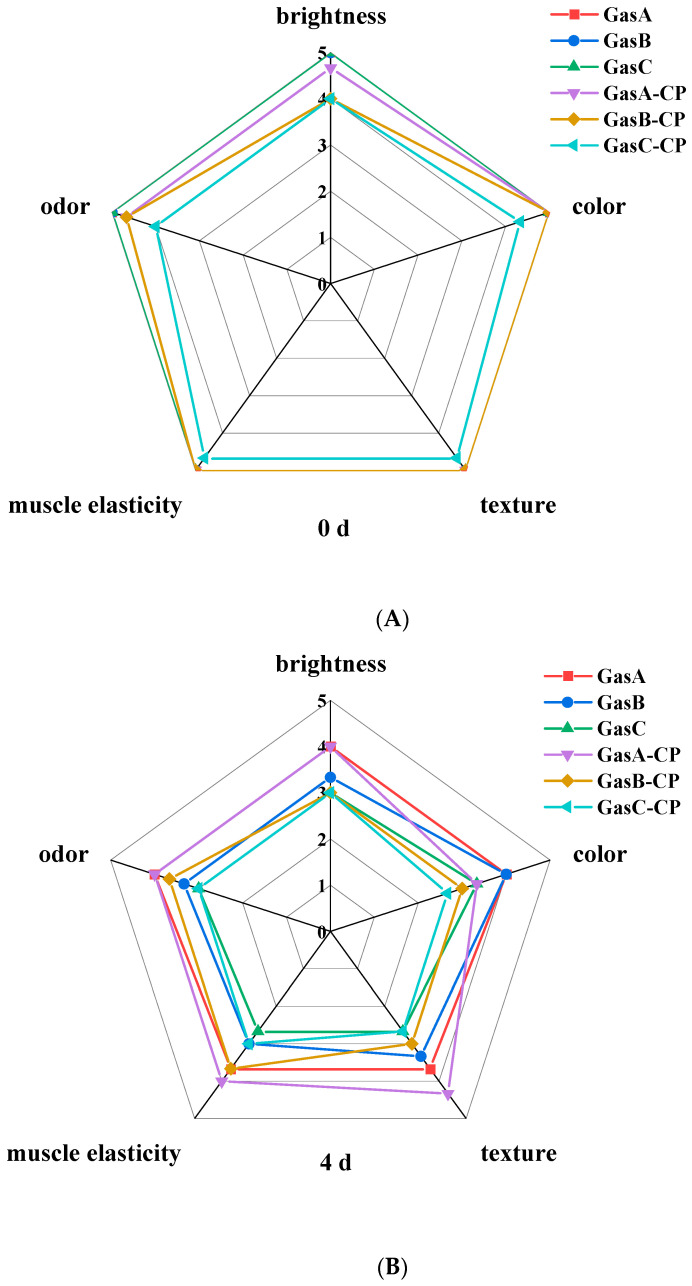

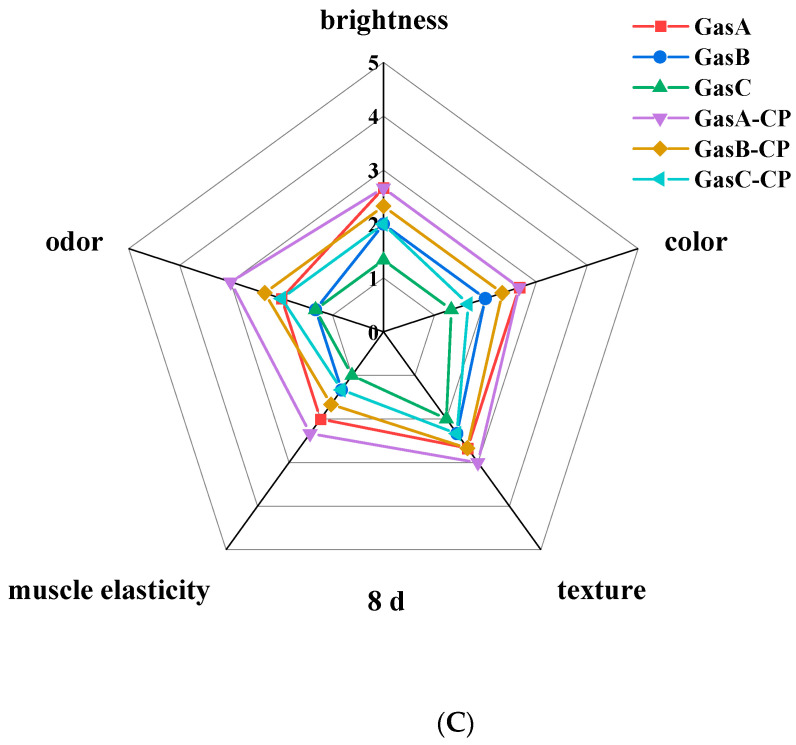
Sensory evaluation of tilapia fillets under different treatment conditions during refrigerator storage ((**A**): 0 days, (**B**): 4 days, (**C**): 8 days). CP: The treatment voltage is 70 kV, and the treatment time is 3 min.

Sensory analysis is an important index to describe and judge the acceptability of food quality. As shown in Figure 1, at 0 d, the scores of the CP and untreated groups were both high, indicating that the quality of the tilapia fillets was high. With the extension of refrigerator storage time, the sensory scores of the CP and untreated groups decreased gradually. At 8 d, the overall scores of the CP groups were higher than those of the untreated groups. Similar studies found that the sensory scores of red shrimp [25] and chub mackerel [26] decreased relatively slowly during refrigerator storage after CP treatment. In addition, the texture, muscle elasticity, odor, etc., of fish in the GasA-CP group were kept in a good state, and the overall quality of the GasA-CP group was obviously superior to other groups and did not exceed that acceptable limit, indicating that low-O_2_ packaging and CP treatment are beneficial to the preservation of fish quality.

### 3.2. Primary Oxidation of Lipid

**Figure 2:**
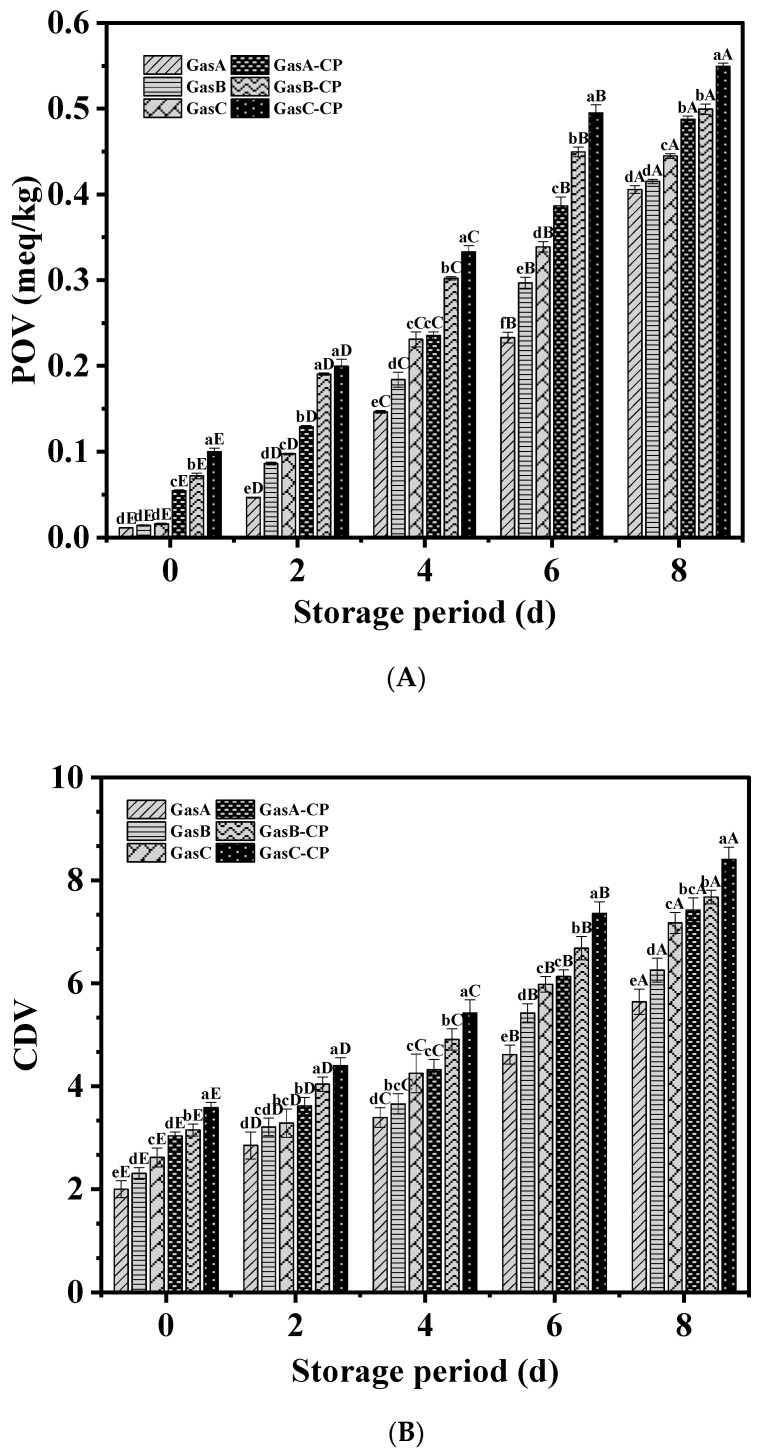
Changes in POV (**A**) and CDV (**B**) of tilapia fillets under different treatment conditions during refrigerator storage. Bars represent standard deviation (*n* = 3). Different uppercase letters (A–E) on the bars within the same treatment indicate significant differences *(p* < 0.05). Different lowercase letters (a–f) on the bars within the same cold storage time indicate significant differences (*p* < 0.05). Caption: See Figure 1.

The POV and CDV values of lipids represent the degree of lipid oxidation in the initial stage of oxidation. As shown in Figure 2A, the POV values of the CP groups were considerably higher than those of the untreated groups at 0 d (*p* < 0.05). The POV values of the CP groups and untreated groups increased with the extension of the refrigerator storage time, regardless of the changes in the gas composition, and equivalent results were also observed in golden pompano [4]. In the untreated groups, the POV values increased with the increase in the O_2_ concentration in the gas compositions; this was related to the ability of O_2_ to promote oxidation [27]. The POV value of the GasA-CP group (10% O_2_) after 8 d storage was 0.49 meq/kg, which was significantly lower than that of the GasC-CP group (30% O_2_) (*p* < 0.05). This shows that the production of hydroperoxide may have been inhibited after the tilapia fillets were treated with CP under the gas composition with a low O_2_ concentration and that the increase in the POV values then slowed down [8]. In a study by Shiekh and Benjakul [11], it was also found that after Pacific white shrimp were treated with dielectric barrier discharge for 10 min when the concentration of O_2_ was low, the secretion of fine extracellular enzymes, microbial enzymes, and other substances that produce hydroperoxide was restricted, and the POV values were reduced.

The CDV and POV values in the present study exhibited a similar trend (Figure 2B); at the end of refrigerator storage, the lowest CDV value was 7.43 in the GasA-CP group. In the oxidation process of olive oil, the POV and CDV values also showed a good correlation [28].

The ROS and RNS produced after CP treatment mainly originated from O_2_ and N_2_. However, it is generally believed that ROS may be the key to inducing lipid oxidation in food when O_2_ exists in a gas composition for the generation of CP plasma [29]. According to Keener et al. [30], when different gas compositions are used in CP treatment, increasing the O_2_ concentration leads to an increase in the content of ROS, such as ozone, which accelerates the oxidation of lipids by generating peroxide. Similarly, investigations of the application of CP treatment on beef and dairy products have indicated that ROS may be responsible for lipid oxidation [31]. Additionally, RNS generated by N_2_ possibly increase oxidation. Nevertheless, the result of N_2_ production depends on the change in the O_2_ concentration [8]. Therefore, the O_2_ concentration plays an important role in the lipid oxidation caused by the CP treatment of tilapia fillets.

### 3.3. Secondary Oxidation of Lipid

**Figure 3:**
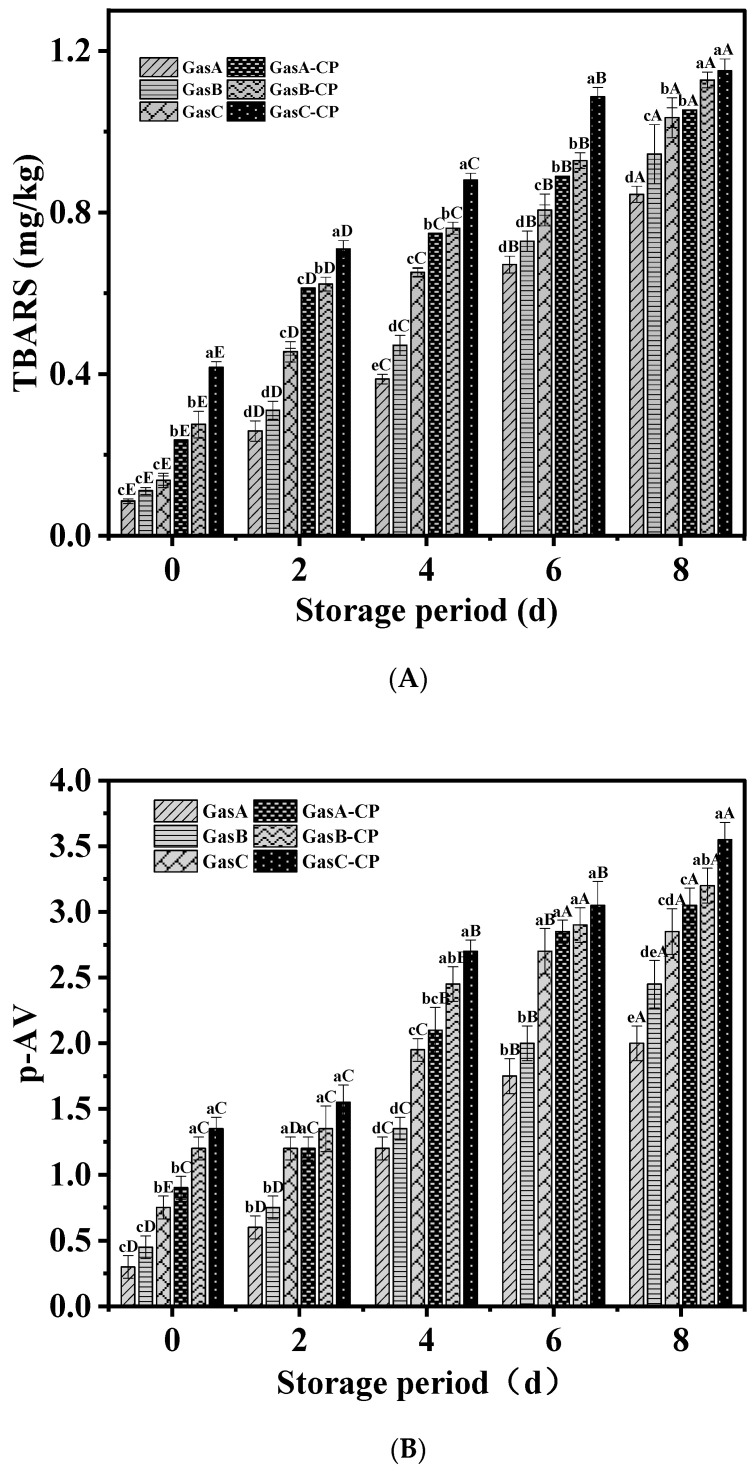

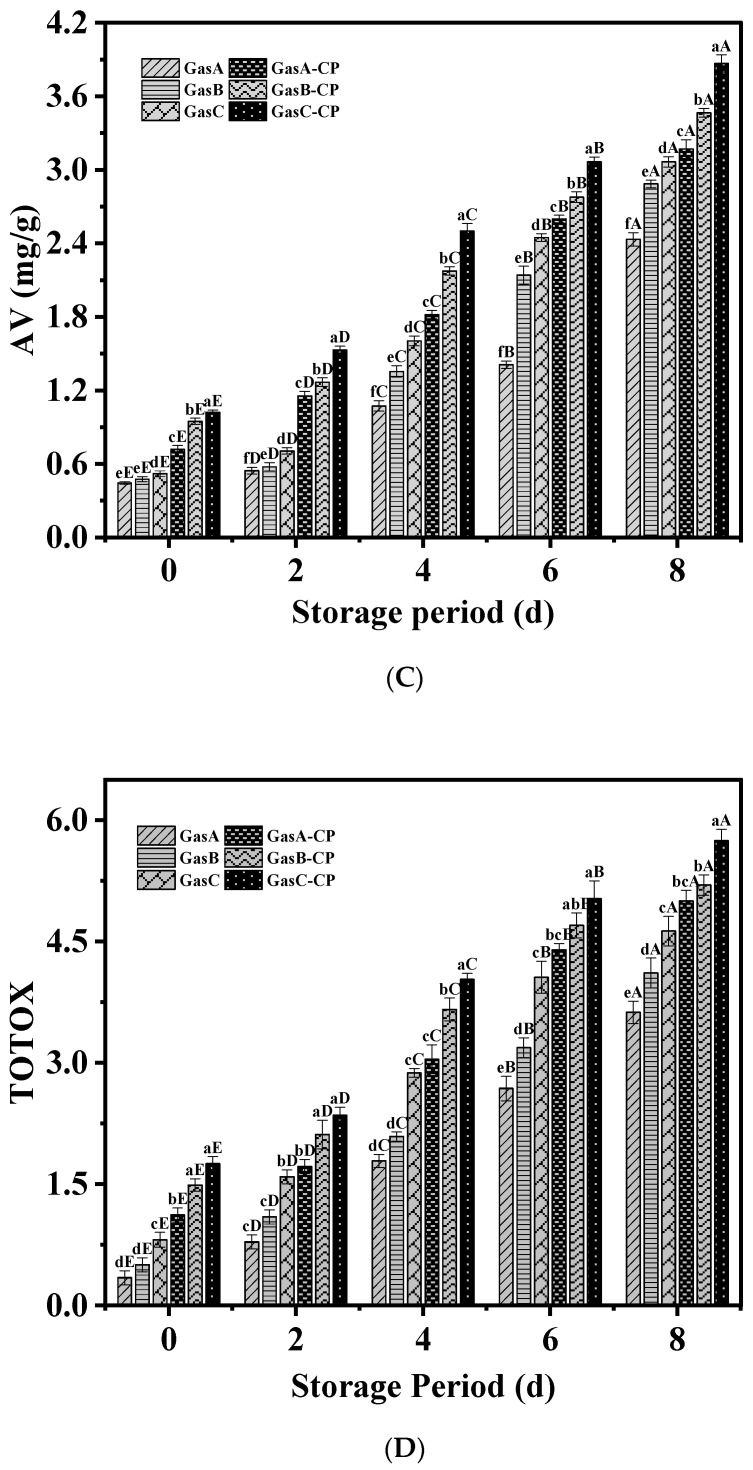
Changes in TBARS (**A**), *p*-AV (**B**), AV (**C**), and TOTOX (**D**) values of tilapia fillets under different treatment conditions during refrigerator storage. Bars represent standard deviation (*n* = 3). Different uppercase letters (A–E) on the bars within the same treatment indicate significant differences (*p* < 0.05). Different lowercase letters (a–f) on the bars within the same cold storage time indicate significant differences (*p* < 0.05). Caption: See Figure 1.

The secondary oxidation products of lipids mainly include acids, alkanes, olefins, aldehydes, alcohols, and ketones. As depicted in Figure 3A, the TBARS values of all groups increased with the extension of the refrigerator storage time, regardless of the changes in the gas composition, and the TBARS values in the treated groups were always higher than those in the untreated groups. With an increase in the O_2_ concentration in the gas composition, the TBARS values in the untreated groups increased. The TBARS values in the CP groups also increased with the concentration of O_2_, with the highest value of 1.15 mg/kg being found in the GasC-CP group after 8 d of refrigerator storage; however, this did not exceed the limit of the TBARS value, which is 2.5 mg/kg [32]. Similar results were reported by Huang et al. [33] in pork after CP treatment.

The *p*-AV values of the CP groups were higher than those of the untreated groups (Figure 3B), which indicated that the content of α-unsaturated and β-unsaturated aldehydes increased. This was because the existence of hydroxyl radicals containing plasma-reactive species accelerated lipid oxidation [34]. The changing trends in both the *p*-AV and AV values were consistent with the TBARS values (Figure 3B,C).

The AV value of the GasA-CP group was 3.17 mg/g at 8 d, which was substantially lower than that of the other two CP groups (*p* < 0.05). Niveditha et al. [34] also discovered that both the *p*-AV and AV values in palm oil increase with longer storage times after CP treatment.

The TOTOX value provides a better assessment of the progressive oxidative deterioration of lipids by considering the total oxidation changes in lipid oxidation. Compared to the untreated groups, the degree of lipid oxidation in the CP groups was significantly higher (*p* < 0.05), as shown in Figure 3D. The lowest TOTOX value was found in the GasA-CP group, which meant that the lower O_2_ concentration facilitated a delay in the oxidation of lipids.

ROS can react with many unsaturated fatty acids, such as oleic acid, to form lipid hydroperoxide, which can further decompose into compounds such as aldehydes and ketones to accelerate the formation of secondary oxidation products [7]. It was found that increasing the O_2_ concentration in the gas could effectively promote the formation of ozone during the CP treatment [35]. Ozone can act on unsaturated bonds in lipids to form ozonides, which are very unstable and easily form aldehydes and other compounds [36]. O_3_ is the main factor causing the increase in the TBARS values in golden pompano after CP treatment [37]. It was found that the secondary oxidation values of lipids were increased in DBD-treated pork with 60% O_2_ packaging, as the high concentration of O_2_ generated more O_3_, which reacts with the lipids in samples and promotes the production of secondary oxidation products [33]. Therefore, a significant difference in the secondary oxidation was found among the CP-treated tilapia fillets with different O_2_ concentrations.

### 3.4. Fatty Acid

A total of eighteen fatty acids were identified in all groups, where the contents of palmitic acid (C16:0), stearic acid (C18:0), oleic acid (C18:1), and linoleic acid (C18:2n-6) were the highest (Table 1). Palmitic acid is the most abundant saturated fatty acid, and compared with the untreated groups, it displayed an apparent upward trend in the CP group during refrigerator storage. The percentages of stearic acid decreased in all groups after 8 d of refrigerator storage; it is speculated that stearic acid may be converted into oleic acid due to the action of oxygen free radicals [8]. Other saturated fatty acids, such as lauric acid (C12:0), are related to cholesterol, and arachidic acid (C20:0) has a low lipid absorption rate [38]; the upward trend is not significant after CP treatment. After 8 d of refrigerator storage, the percentages of total saturated fatty acids in the untreated groups increased with the increase in the O_2_ concentration, and the percentages of total saturated fatty acids in the CP groups were also the lowest under hypoxic packaging. It may be that hypoxia facilitates a delay in the increase in saturated fatty acids.

The total percentages of unsaturated fatty acids in tilapia fillets decreased after CP treatment. The highest decrease in monounsaturated fatty acids was observed for oleic acid; it is speculated that oleic acid is easily decomposed by the reactive free radicals generated by CP to produce hydroperoxide, which is further converted into secondary oxidation products such as aldehydes, ketones, and acids [8]. Although stearic acid can be dehydrogenated into oleic acid by ROS, oleic acid can revert to stearic acid via a reversible reaction with ·H and the existence of ·OH [39]. In addition, the biological hydrogenation of linoleic acid also leads to an increase in stearic acid [40]. Therefore, the percentages of oleic acid in each group decreased after 8 d of refrigerator storage. However, the percentages of palmitoleic acid (C16:1) increased during refrigerator storage, which meant that there were no obvious changes in the percentages of total monounsaturated fatty acids.

After 8 d of refrigerator storage, compared to the untreated groups, the total percentage of polyunsaturated fatty acids in the treated samples decreased; the highest decrease in value was 13.4%, which was found in the GasC-CP groups. O_2_ can induce the decomposition of polyunsaturated fatty acids to form short-chain saturated fatty acids and low molecular compounds [41]. Higher O_2_ can promote the formation of ROS, such as ozone and hydrogen peroxide, during CP treatment [29]; in addition, ROS can react with carbon double bonds to generate aldehydes and carboxylic acids [42], accelerating the decomposition of polyunsaturated fatty acids. In our study, linoleic acid, as the most abundant polyunsaturated fatty acid, decreased by 3.02% in the GasC-CP group but was 2.58% in the GasA-CP group. During refrigerator storage, linoleic acid and α-linolenic acid (18:3n-3), containing two and three double bonds, are sensitive to ROS and are converted into saturated fatty acids [29]. Afshar et al. [43] reported that the polyunsaturated fatty acids C18:3 and C18:2 are rapidly decomposed by the free radicals generated during CP treatment and decrease significantly in the later storage period, whereas saturated fatty acids increase with an increasing storage time. After 8 d of storage, the percentages of eicosapentaenoic acid (EPA, C20:5n-3) and docosahexaenoic acid (DHA, C22:6n-3) in the GasA-CP group were 0.54% and 0.67%, respectively; however, in the GasC-CP group, they were 0.54% and 0.67%, respectively. Similarly, the EPA and DHA of Pacific white shrimp can be kept at a high level after being treated with low O_2_ dielectric barrier discharge and refrigerated for 15 d [11]. What is more, the percentages of n-3 and n-6 fatty acids in the GasA-CP group were 3.64% and 26.3%, respectively; these are higher than those in the other two CP groups. Therefore, the CP treatment enhanced the decomposition of polyunsaturated fatty acids in tilapia fillets with high O_2_ packaging.

The alteration in the fatty acid concentration is attributable to the oxidation of unsaturated fatty acids, wherein their double bonds are decomposed by the reactive species produced during the CP treatment. ROS primarily target the methyl groups in lipid moieties and display a greater affinity for those linked by double bonds; this is due to the fact that the energy needed for the abstraction of a hydrogen atom is significantly lower than the CH-bonds linked elsewhere (272 kJ/mol and 422 kJ/mol) [29]. As a result, the double bonds in fatty acids are easily induced by ROS. Jadhav and Annapure [7] found that a low concentration of O_2_ has a slight effect on lipid oxidation in meat. Therefore, controlling the O_2_ concentration in CP excite gas may be an effective measure by which to limit the decline in unsaturated fatty acids and the increase in saturated fatty acids.

### 3.5. Pearson’s Correlation Coefficients

**Figure 4:**
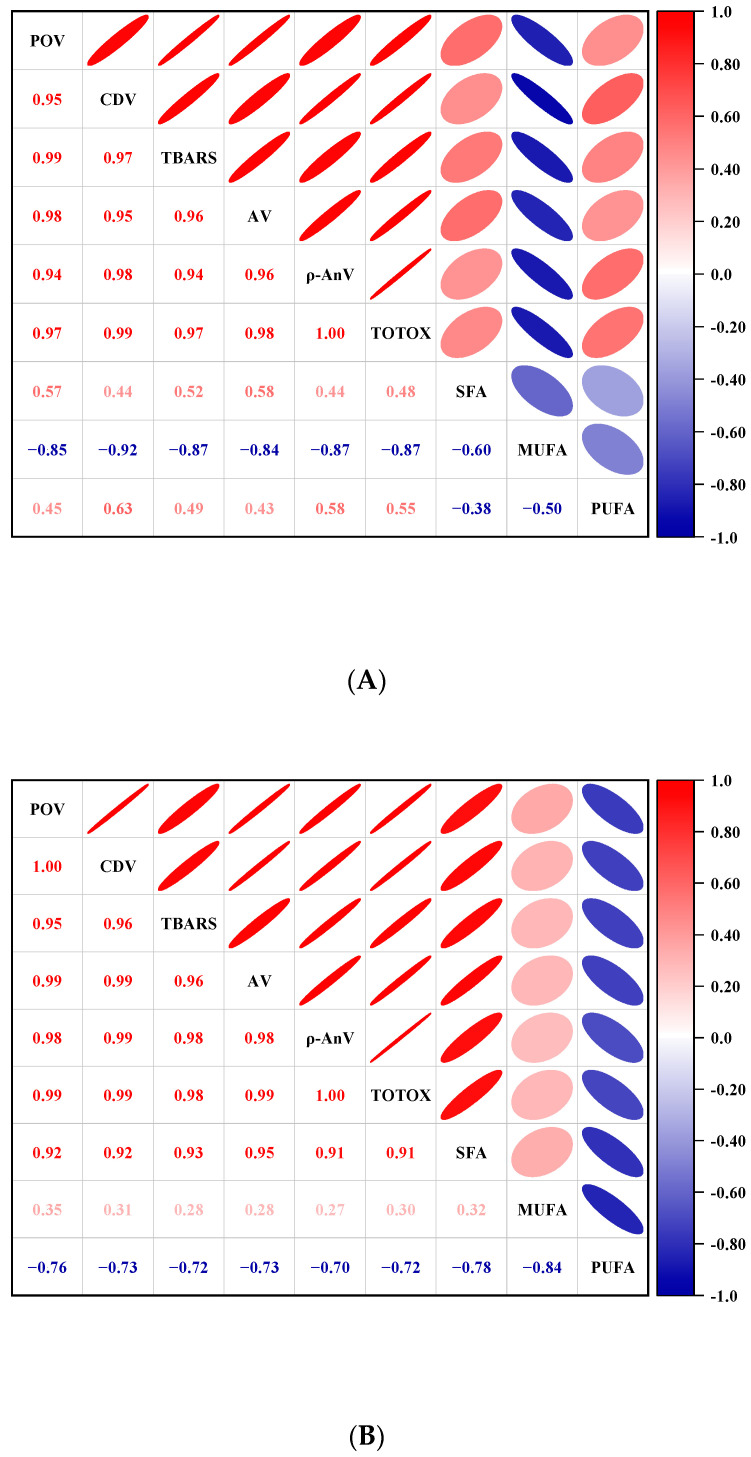

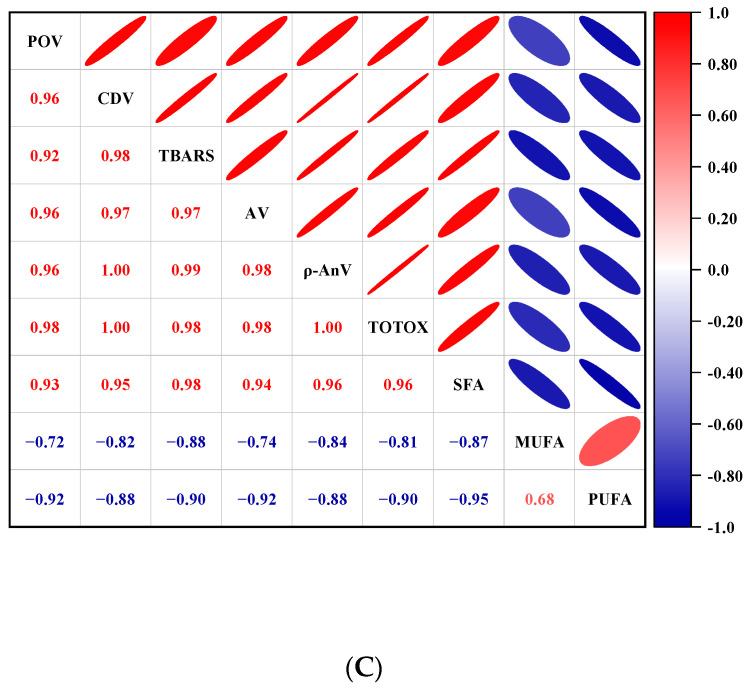
Correlation analysis of lipid oxidation value and fatty acids ((**A**): 0 days, (**B**): 4 days, (**C**): 8 days).

Pearson’s correlation coefficients for the primary and secondary oxidation values for the lipids during refrigerator storage are shown in Figure 4; both exceed 0.9, showing a significant positive correlation (*p* < 0.01).

At 0 d, all lipid oxidation values exhibited a negative correlation, but only with monounsaturated fatty acids (*p* < 0.05). The correlation coefficient of the CDV value and monounsaturated fatty acids was 0.92, which is higher than the other lipid oxidation values. It can be concluded that in the early stage of refrigerator storage, monounsaturated fatty acids are mainly decomposed, which leads to the formation of primary oxidation product hydroperoxide [8]. After 4 d, the primary oxidation value and secondary oxidation value of the lipids were positively correlated with saturated fatty acids (*p* < 0.05). The correlation coefficients of the POV value, CDV value, TBARS value, and AV value with saturated fatty acids were 0.92, 0.92, 0.93, and 0.95, respectively, which showed a stronger positive correlation than the *p*-AV value and TOTOX value (*p* < 0.01). After 8 d, the primary oxidation value and secondary oxidation value of the lipids were positively correlated with the percentages of saturated fatty acids (*p* < 0.01) and negatively correlated with the percentages of monounsaturated fatty acids and polyunsaturated fatty acids (*p* < 0.05). The existence of O_2_ and CP treatment can promote the decomposition of unsaturated fatty acids and produce some short-chain saturated fatty acids, as well as oxidation products such as hydroperoxide, aldehyde, and alcohol [29,41]; this makes the unsaturated fatty acid concentration decrease and the lipid oxidation value and saturated fatty acid concentration increase.

In this study, with the extension of refrigerator storage, the correlation coefficient of lipid oxidation values and fatty acid content of tilapia fillets increased. On the basis of correlation, the oxidation process of lipids can be better reflected by the change in the fatty acid content.

## 4. Conclusions

Tilapia fillets with 10% O_2_ packaging showed a lower degree of lipid oxidation after CP treatment, as well as the highest sensory score. The values of both the primary and secondary lipid oxidation factors decreased as the O_2_ concentration reduced during refrigerator storage. The lowest percentages of saturated fatty acids were found in the GasA-CP group; conversely, the monounsaturated fatty acid percentages basically did not display any notable change in all groups. The percentages of polyunsaturated (n-3 and n-6) fatty acids in the GasA-CP group were reduced less than those in the other CP treatment groups. Pearson’s correlation analysis revealed that the POV value, CDV value, TBARS value, AV value, *p*-AV value, and TOTOX value were positively and negatively correlated with the changes in the percentages of saturated and unsaturated fatty acids, respectively.

Therefore, controlling the gas composition could decelerate lipid oxidation in CP-treated tilapia fillets and simultaneously reduce the oxidational decomposition of unsaturated fatty acids. This reasonably preserves the nutritional quality and normal flavor of tilapia fillets. This investigation could provide a theoretical reference for the application of CP in fish and aquatic product preservation.

## Figures and Tables

**Table 1:** Fatty acid percentage of tilapia fillets under different treatment conditions during refrigerator storage.

Fatty Acid (%)	Days	GasA	GasB	GasC	GasA-CP	GasB-CP	GasC-CP
C12:0	0	0.47 ± 0.04 ^aB^	0.44 ± 0.09 ^aC^	0.36 ± 0.09 ^aB^	0.35 ± 0.04 ^aB^	0.29 ± 0.02 ^aC^	0.52 ± 0.13 ^aC^
	4	0.38 ± 0.07 ^cB^	0.90 ± 0.03 ^aB^	0.37 ± 0.05 ^cB^	0.45 ± 0.04 ^cB^	0.60 ± 0.06 ^bB^	0.83 ± 0.04 ^aB^
	8	1.23 ± 0.05 ^bA^	1.37 ± 0.04 ^abA^	1.29 ± 0.14 ^bA^	1.60 ± 0.08 ^aA^	0.98 ± 0.06 ^cA^	1.15 ± 0.05 ^bcA^
C14:0	0	2.18 ± 0.15 ^abA^	2.21 ± 0.24 ^abA^	1.89 ± 0.15 ^bB^	2.37 ± 0.19 ^abB^	2.30 ± 0.07 ^abB^	2.73 ± 0.28 ^aB^
	4	2.34 ± 0.11 ^bA^	2.82 ± 0.39 ^abA^	2.39 ± 0.09 ^abB^	2.75 ± 0.14 ^abB^	2.92 ± 0.20 ^abA^	3.02 ± 0.07 ^aAB^
	8	2.62 ± 0.13 ^bA^	3.07 ± 0.10 ^bA^	3.40 ± 0.20 ^aA^	3.22 ± 0.21 ^aA^	3.30 ± 0.16 ^aA^	3.43 ± 0.15 ^aA^
C15:0	0	1.08 ± 0.14 ^cB^	2.02 ± 0.28 ^bA^	2.65 ± 0.14 ^aA^	1.78 ± 0.13 ^bA^	2.21 ± 0.06 ^abA^	2.04 ± 0.22 ^bA^
	4	1.97 ± 0.23 ^aA^	1.01 ± 0.24 ^bB^	2.45 ± 0.21 ^aA^	1.87 ± 0.22 ^aA^	2.13 ± 0.08 ^aA^	1.96 ± 0.15 ^aA^
	8	1.60 ± 0.13 ^abAB^	1.86 ± 0.11 ^aB^	1.18 ± 0.08 ^cB^	1.65 ± 0.15 ^abA^	1.46 ± 0.01 ^bcB^	1.54 ± 0.12 ^abA^
C16:0	0	24.80 ± 0.17 ^aA^	24.40 ± 0.61 ^abB^	19.92 ± 0.64 ^dB^	23.70 ± 0.02 ^bcB^	23.81 ± 0.27 ^abB^	24.66 ± 0.11 ^cB^
	4	24.64 ± 0.14 ^bA^	26.27 ± 0.34 ^aA^	24.82 ± 0.20 ^cA^	26.12 ± 0.25 ^aA^	24.57 ± 0.11 ^bB^	26.58 ± 0.25 ^aA^
	8	24.22 ± 0.55 ^cA^	24.84 ± 0.10b ^cB^	26.07 ± 0.62 ^cA^	26.60 ± 0.37 ^bA^	28.00 ± 0.16 ^abA^	27.11 ± 0.46 ^aA^
C17:0	0	1.15 ± 0.27 ^bB^	1.85 ± 0.14 ^abA^	2.54 ± 0.22 ^aA^	1.48 ± 0.38 ^bA^	1.90 ± 0.11 ^abB^	1.87 ± 0.09 ^abA^
	4	1.79 ± 0.20 ^bAB^	0.66 ± 0.11 ^cB^	1.99 ± 0.11 ^abA^	1.54 ± 0.21 ^bA^	2.38 ± 0.10 ^aA^	1.98 ± 0.22 ^abA^
	8	2.31 ± 0.20 ^aA^	1.92 ± 0.26 ^abcA^	1.40 ± 0.13 ^cB^	2.16 ± 0.29 ^abA^	1.62 ± 0.06 ^bcB^	1.72 ± 0.14 ^abcA^
C18:0	0	5.17 ± 0.18 ^cA^	6.09 ± 0.04 ^abA^	6.48 ± 0.20 ^aA^	5.69 ± 0.31 ^bcA^	6.09 ± 0.12 ^abA^	5.15 ± 0.11 ^cA^
	4	5.35 ± 0.24 ^aA^	5.41 ± 0.56 ^aA^	5.31 ± 0.07 ^aB^	5.33 ± 0.26 ^aA^	5.15 ± 0.24 ^aB^	4.73 ± 0.16 ^aA^
	8	4.80 ± 0.09 ^bcA^	4.98 ± 0.22 ^bcA^	5.59 ± 0.26 ^aB^	4.38 ± 0.16 ^cB^	4.94 ± 0.12 ^bcB^	5.16 ± 0.23 ^abA^
C20:0	0	0.90 ± 0.01 ^aA^	0.67 ± 0.06 ^bA^	0.87 ± 0.05 ^aA^	0.87 ± 0.1 ^aA^	0.78 ± 0.01 ^abB^	0.77 ± 0.02 ^abB^
	4	1.20 ± 0.27 ^aA^	0.97 ± 0.19 ^aA^	0.87 ± 0.13 ^aA^	0.95 ± 0.09 ^aA^	0.89 ± 0.04 ^aAB^	0.81 ± 0.09 ^aB^
	8	1.17 ± 0.07 ^aA^	0.81 ± 0.02 ^cdA^	0.93 ± 0.06 ^bcA^	0.70 ± 0.05 ^dA^	0.99 ± 0.08 ^abA^	1.08 ± 0.04 ^abA^
C22:0	0	0.70 ± 0.17 ^bA^	0.81 ± 0.16^bA^	1.24 ± 0.11 ^aA^	0.84 ± 0.11 ^bA^	0.73 ± 0.03 ^bAB^	0.65 ± 0.07 ^bA^
	4	0.67 ± 0.12 ^abA^	1.08 ± 0.26^aA^	0.88 ± 0.08 ^abB^	0.66 ± 0.12 ^abA^	1.02 ± 0.14 ^abA^	0.56 ± 0.03 ^bA^
	8	0.94 ± 0.07 ^aA^	0.65 ± 0.08 ^bA^	0.51 ± 0.02 ^bC^	0.58 ± 0.04 ^bA^	0.66 ± 0.06 ^bB^	0.62 ± 0.04 ^bA^
ΣSFA	0	36.65 ± 0.77 ^cdA^	38.49 ± 0.30 ^aA^	35.95 ± 0.22 ^dB^	37.08 ± 0.55 ^bcdB^	38.44 ± 0.19 ^abcA^	38.39 ± 0.13 ^abcC^
	4	38.34 ± 0.84 ^bcA^	39.13 ± 0.83 ^abA^	39.08 ± 0.27 ^cB^	39.68 ± 0.82 ^abA^	39.66 ± 0.24 ^abB^	40.47 ± 0.22 ^aB^
	8	38.91 ± 0.57 ^dA^	39.51 ± 0.29 ^cdA^	40.36 ± 0.49 ^bcA^	40.90 ± 0.12 ^abA^	41.95 ± 0.38 ^aA^	41.80 ± 0.38 ^aA^
C16:1	0	3.44 ± 0.26 ^aB^	3.23 ± 0.14 ^aA^	2.84 ± 0.20 ^aB^	3.29 ± 0.28 ^aB^	2.85 ± 0.13 ^aB^	3.41 ± 0.16 ^aA^
	4	3.79 ± 0.09 ^aAB^	3.79 ± 0.44 ^aA^	3.92 ± 0.33 ^aA^	3.60 ± 0.06 ^aAB^	3.54 ± 0.25 ^aA^	3.73 ± 0.16 ^aA^
	8	4.14 ± 0.12 ^aA^	4.00 ± 0.54 ^aA^	4.03 ± 0.36 ^aA^	4.27 ± 0.36 ^aA^	3.55 ± 0.11 ^aA^	3.67 ± 0.25 ^aA^
C18:1	0	28.75 ± 0.53 ^aA^	26.98 ± 0.54 ^abcA^	27.89 ± 0.63 ^abA^	25.83 ± 0.85 ^cA^	26.54 ± 0.19 ^bcAB^	25.12 ± 0.65 ^bcA^
	4	24.45 ± 0.41 ^bC^	26.40 ± 0.18 ^aA^	26.55 ± 0.60 ^aA^	25.32 ± 0.32 ^abA^	26.06 ± 0.15 ^aB^	25.99 ± 0.47 ^aA^
	8	26.41 ± 0.33 ^aB^	26.04 ± 0.23 ^aA^	25.51 ± 0.38 ^bA^	24.92 ± 0.49 ^bA^	25.55 ± 0.46 ^bA^	25.89 ± 0.74 ^bA^
ΣMUFA	0	32.19 ± 0.36 ^aA^	30.21 ± 0.62 ^abA^	30.73 ± 0.80 ^abA^	29.12 ± 1.04 ^bA^	29.39 ± 0.31 ^bA^	28.54 ± 0.57 ^bA^
	4	28.24 ± 0.33 ^cC^	30.19 ± 0.30 ^abA^	30.47 ± 0.33 ^aA^	28.93 ± 0.35 ^bcA^	29.60 ± 0.33 ^abA^	29.73 ± 0.58 ^abA^
	8	30.55 ± 0.38 ^aB^	30.05 ± 0.52 ^aA^	29.54 ± 0.09 ^aA^	29.19 ± 0.14 ^aA^	29.10 ± 0.54 ^aA^	29.55 ± 0.70 ^aA^
C18:2n-6	0	24.12 ± 0.55 ^aA^	21.79 ± 0.20 ^bA^	21.91 ± 0.53 ^bB^	24.22 ± 0.19 ^aA^	22.55 ± 0.12 ^bA^	24.52 ± 0.64 ^aA^
	4	23.63 ± 0.09 ^aA^	20.47 ± 0.80 ^cA^	20.40 ± 0.23 ^aA^	22.72 ± 0.58 ^abB^	21.47 ± 0.54 ^bcA^	21.07 ± 0.27 ^cB^
	8	21.69 ± 0.22 ^aB^	22.00 ± 0.32 ^aA^	21.47 ± 0.22 ^abB^	21.64 ± 0.16 ^aB^	21.48 ± 0.12 ^abA^	20.80 ± 0.33 ^cB^
C18:3n-3	0	2.31 ± 0.07 ^aA^	1.74 ± 0.10 ^bA^	1.81 ± 0.03 ^bB^	2.35 ± 0.25 ^aA^	1.93 ± 0.13 ^abAB^	2.15 ± 0.08 ^abA^
	4	2.25 ± 0.32 ^aA^	2.26 ± 0.39 ^aA^	1.98 ± 0.15 ^aAB^	1.94 ± 0.07 ^aA^	1.67 ± 0.11 ^aB^	1.67 ± 0.13 ^aA^
	8	2.46 ± 0.08 ^aA^	1.83 ± 0.01 ^bcA^	2.25 ± 0.10 ^abA^	2.11 ± 0.09 ^abcA^	2.07 ± 0.08 ^abcA^	1.76 ± 0.28 ^cA^
C20:2n-6	0	0.67 ± 0.10 ^aA^	0.84 ± 0.16 ^aA^	0.93 ± 0.09 ^aA^	0.82 ± 0.06 ^aA^	0.79 ± 0.07 ^aA^	0.90 ± 0.20 ^aA^
	4	0.74 ± 0.02 ^bA^	0.97 ± 0.07 ^aA^	1.01 ± 0.05 ^aA^	0.82 ± 0.12 ^abA^	0.72 ± 0.05 ^bA^	0.67 ± 0.03 ^bA^
	8	0.65 ± 0.02 ^bcA^	0.69 ± 0.01 ^bcA^	0.83 ± 0.04 ^aA^	0.59 ± 0.04 ^cA^	0.71 ± 0.03 ^bA^	0.61 ± 0.04 ^bcA^
C20:4n-6	0	2.29 ± 0.22 ^eB^	3.85 ± 0.21 ^bcA^	4.69 ± 0.29 ^aA^	3.30 ± 0.26 ^cdAB^	4.13 ± 0.30 ^abA^	3.00 ± 0.13 ^deA^
	4	3.21 ± 0.18 ^aA^	2.74 ± 0.24 ^aB^	3.73 ± 0.49 ^aA^	3.51 ± 0.10 ^aA^	3.42 ± 0.28 ^aA^	3.02 ± 0.32 ^aA^
	8	2.77 ± 0.13 ^aAB^	2.69 ± 0.06 ^aB^	2.55 ± 0.10 ^abB^	2.83 ± 0.10 ^aB^	2.17 ± 0.22 ^bB^	2.67 ± 0.19 ^aA^
C20:5n-3	0	0.55 ± 0.07 ^aA^	0.63 ± 0.05 ^aB^	0.67 ± 0.06 ^aA^	0.65 ± 0.06 ^aA^	0.59 ± 0.01 ^aA^	0.54 ± 0.05 ^aAB^
	4	0.58 ± 0.09 ^bA^	1.18 ± 0.12 ^aA^	0.57 ± 0.03 ^bA^	0.53 ± 0.03 ^bA^	0.66 ± 0.06 ^bA^	0.64 ± 0.12 ^bA^
	8	0.68 ± 0.11 ^abA^	0.42 ± 0.05 ^bcB^	0.74 ± 0.12 ^aA^	0.54 ± 0.09 ^abcA^	0.43 ± 0.04 ^bcB^	0.33 ± 0.03 ^cB^
C22:4n-6	0	0.73 ± 0.14 ^cB^	1.44 ± 0.16 ^abA^	1.92 ± 0.31 ^aA^	1.29 ± 0.19 ^bcA^	1.31 ± 0.03 ^bcAB^	0.92 ± 0.04 ^bcA^
	4	1.24 ± 0.13 ^abA^	1.07 ± 0.04 ^abA^	1.31 ± 0.03 ^aAB^	0.93 ± 0.06 ^bA^	1.39 ± 0.12 ^aA^	1.05 ± 0.18 ^abA^
	8	1.45 ± 0.06 ^abA^	1.62 ± 0.38 ^aA^	1.09 ± 0.08 ^abB^	1.21 ± 0.01 ^abA^	0.84 ± 0.08 ^bB^	1.15 ± 0.22 ^abA^
C22:5n-3	0	0.30 ± 0.01 ^bB^	0.30 ± 0.02 ^bB^	0.43 ± 0.13 ^abA^	0.40 ± 0.07 ^abA^	0.28 ± 0.02 ^bA^	0.54 ± 0.09 ^aA^
	4	0.50 ± 0.05 ^bA^	0.94 ± 0.14 ^aA^	0.35 ± 0.05 ^bA^	0.26 ± 0.03 ^bA^	0.41 ± 0.08 ^bA^	0.43 ± 0.06 ^bA^
	8	0.30 ± 0.05 ^bB^	0.57 ± 0.07 ^aB^	0.46 ± 0.13 ^abA^	0.32 ± 0.01 ^bA^	0.37 ± 0.02 ^abA^	0.41 ± 0.02 ^abA^
C22-6n-3	0	0.64 ± 0.09 ^cdB^	0.70 ± 0.06 ^cdB^	0.96 ± 0.04 ^abB^	0.80 ± 0.08 ^bcA^	1.10 ± 0.08 ^aA^	0.50 ± 0.07 ^dC^
	4	1.28 ± 0.04 ^aA^	1.04 ± 0.09 ^aA^	1.11 ± 0.08 ^aB^	0.68 ± 0.08 ^bA^	0.99 ± 0.15 ^aA^	1.24 ± 0.09 ^aA^
	8	0.54 ± 0.02 ^cB^	0.62 ± 0.08 ^bB^	0.71 ± 0.10 ^abA^	0.67 ± 0.09 ^bA^	0.63 ± 0.04 ^bB^	0.94 ± 0.06 ^aB^
ΣPUFA	0	31.85 ± 0.71 ^abAB^	31.29 ± 0.39 ^bA^	33.32 ± 0.99 ^abA^	33.83 ± 0.61 ^aA^	32.50 ± 0.26 ^abA^	33.07 ± 0.51 ^abA^
	4	33.59 ± 0.61 ^aA^	30.69 ± 0.68 ^bA^	30.45 ± 0.34 ^aA^	31.39 ± 0.80 ^bB^	30.74 ± 0.56 ^bB^	29.80 ± 0.38 ^bB^
	8	30.54 ± 0.40 ^aB^	30.45 ± 0.31 ^aA^	30.09 ± 0.42 ^aB^	29.91 ± 0.11 ^aB^	28.70 ± 0.32 ^bC^	28.65 ± 0.43 ^bB^
Σn-3PUFA	0	3.80 ± 0.03 ^abB^	3.37 ± 0.15 ^bB^	3.87 ± 0.44 ^aA^	4.19 ± 0.23 ^aA^	3.90 ± 0.07 ^abA^	3.73 ± 0.15 ^abA^
	4	4.60 ± 0.23 ^bA^	5.43 ± 0.35 ^aA^	4.00 ± 0.16 ^bcB^	3.41 ± 0.18 ^cB^	3.74 ± 0.25 ^cAB^	3.98 ± 0.24 ^bcA^
	8	3.99 ± 0.14 ^bB^	3.44 ± 0.19 ^bcB^	4.85 ± 0.23 ^aA^	3.64 ± 0.21 ^bcAB^	3.30 ± 0.09 ^cB^	3.44 ± 0.21 ^bcA^
Σn-6PUFA	0	28.10 ± 0.54 ^aA^	27.92 ± 0.41 ^aA^	29.45 ± 0.78 ^aA^	29.64 ± 0.50 ^aA^	28.60 ± 0.27 ^aA^	29.34 ± 0.60 ^aA^
	4	28.81 ± 0.35 ^aA^	25.30 ± 0.66 ^dB^	26.46 ± 0.29 ^cdB^	27.98 ± 0.71 ^cdB^	27.00 ± 0.31 ^bcB^	25.82 ± 0.20 ^cdB^
	8	26.56 ± 0.27 ^aB^	27.00 ± 0.12 ^aA^	25.34 ± 0.23 ^bB^	26.27 ± 0.26 ^bB^	25.40 ± 0.36 ^bC^	25.21 ± 0.35 ^bB^
Σn-3/Σn-6PUFA	0	0.14 ± 0.00 ^aA^	0.12 ± 0.01 ^bB^	0.13 ± 0.00 ^abB^	0.14 ± 0.01 ^abA^	0.14 ± 0.00 ^aA^	0.13 ± 0.01 ^abA^
	4	0.16 ± 0.01 ^bA^	0.21 ± 0.02 ^aA^	0.15 ± 0.01 ^bB^	0.12 ± 0.01 ^cA^	0.14 ± 0.01 ^bcA^	0.15 ± 0.01 ^bA^
	8	0.15 ± 0.00 ^bA^	0.13 ± 0.01 ^bB^	0.19 ± 0.01 ^aA^	0.14 ± 0.01 ^bA^	0.13 ± 0.00 ^bA^	0.14 ± 0.01 ^bA^

Standard deviation (*n* = 3). Different capital letters (A–C) in the same column indicate significant differences (*p* < 0.05). Different lowercase letters (a–e) on the bars within the same storage time indicate significant differences (*p* < 0.05). Caption: See Figure 1.

## Data Availability

Data are contained within the article or Supplementary Material.

## Supplementary Materials

The following supporting information can be downloaded at: https://www.mdpi.com/article/10.3390/foods13010165/s1, Table S1: The sensory evaluation standard of the tilapia fillets.
