# Supplementary material for: Effects of Gas Composition on the Lipid Oxidation and Fatty Acid Concentration of Tilapia Fillets Treated with In-Package Atmospheric Cold Plasma
Source: Foods. 2024 Jan 3;13(1):165. doi: 10.3390/foods13010165 (PMC10779136; doi:10.3390/foods13010165)
Supplement: Supplementary file 1 [file foods-13-00165-s001.zip › foods-2776868-supplementary.pdf]

Table S1. The sensory evaluation standard of the tilapia fillets.

Sensory Characteristics	Score				
	**5**	**4**	**3**	**2**	**1**
Brightness	Very brigh	Bright	Slightly dim	Partially dim	Dim
Color	Very nice bright white	Bright white	White	Slightly gray	Grayish white, partially yellow
Texture	Complete, the texture is very clear	Compact, the texture is clear	Not tight, the texture is clear	Not tight, the texture is loose	Not tight, the texture is completely loose
Muscle elasticity	Tight, very good elasticity	Tight, elasticity	Little soft, insufficient elasticity	Soft, poor elasticity	Loose, elasticity disappears
Odor	No peculiar odor, very light fishy smell	No peculiar odor, fishy smell	No peculiar odor, strong fishy smell	Slightly peculiar odor, rich fishy smell	Strong odor, foul smell
